# Metabolomics for the design of new metabolic engineering strategies for improving aerobic succinic acid production in *Escherichia coli*

**DOI:** 10.1007/s11306-022-01912-9

**Published:** 2022-07-20

**Authors:** Antonio Valle, Zamira Soto, Howbeer Muhamadali, Katherine A. Hollywood, Yun Xu, Jonathan R. Lloyd, Royston Goodacre, Domingo Cantero, Gema Cabrera, Jorge Bolivar

**Affiliations:** 1 Department of Biomedicine, Biotechnology and Public Health-Biochemistry and Molecular Biology, University of Cadiz, Campus Universitario de Puerto Real, 11510 Puerto Real, Cádiz, Spain; 2 Department of Chemical Engineering and Food Technology, University of Cadiz, Campus Universitario de Puerto Real, 11510 Puerto Real, Cádiz, Spain; 3 Faculty of Basic and Biomedical Sciences, Universidad Simón Bolívar, 080020 Barranquilla, Colombia; 4 School of Chemistry, Manchester Institute of Biotechnology, University of Manchester, Manchester, M1 7DN UK; 5 Department of Biochemistry and Systems Biology, Institute of Integrative Systems, Molecular and Integrative Biology, University of Liverpool, Biosciences Building, Crown Street, Liverpool, L69 7ZB UK; 6 Manchester Centre for Synthetic Biology of Fine and Speciality Chemicals (SYNBIOCHEM), Manchester Institute of Biotechnology, The University of Manchester, Manchester, M1 7DN UK; 7 Williamson Research Centre, School of Earth & Environmental Sciences, University of Manchester, Manchester, M13 9PL UK; 8 Institute of Viticulture and Agri-Food Research (IVAGRO) - International Campus of Excellence (ceiA3), University of Cadiz, 11510 Puerto Real, Cádiz, Spain; 9 Institute of Biomolecules (INBIO), University of Cadiz, 11510 Puerto Real, Cádiz, Spain

**Keywords:** *Escherichia coli*, Metabolomics, Succinic acid, TCA cycle, GC–MS and mannitol dehydrogenase (MtlD)

## Abstract

**Introduction:**

Glycerol is a byproduct from the biodiesel industry that can be biotransformed by *Escherichia coli* to high added-value products such as succinate under aerobic conditions. The main genetic engineering strategies to achieve this aim involve the mutation of succinate dehydrogenase (*sdhA*) gene and also those responsible for acetate synthesis including acetate kinase, phosphate acetyl transferase and pyruvate oxidase encoded by *ackA*, *pta* and *pox* genes respectively in the *ΔsdhAΔack-ptaΔpox* (M4) mutant. Other genetic manipulations to rewire the metabolism toward succinate consist on the activation of the glyoxylate shunt or blockage the pentose phosphate pathway (PPP) by deletion of isocitrate lyase repressor (*iclR*) or gluconate dehydrogenase (*gnd*) genes on M4-*ΔiclR* and M4-*Δgnd* mutants respectively.

**Objective:**

To deeply understand the effect of the blocking of the pentose phosphate pathway (PPP) or the activation of the glyoxylate shunt*,* metabolite profiles were analyzed on M4-*Δgnd*, M4-*ΔiclR* and M4 mutants.

**Methods:**

Metabolomics was performed by FT-IR and GC–MS for metabolite fingerprinting and HPLC for quantification of succinate and glycerol.

**Results:**

Most of the 65 identified metabolites showed lower relative levels in the M4-*ΔiclR* and M4-*Δgnd* mutants than those of the M4. However, fructose 1,6-biphosphate, trehalose, isovaleric acid and mannitol relative concentrations were increased in M4-*ΔiclR* and M4-*Δgnd* mutants. To further improve succinate production, the synthesis of mannitol was suppressed by deletion of mannitol dehydrogenase (*mtlD*) on M4-*ΔgndΔmtlD* mutant that increase ~ 20% respect to M4-*Δgnd*.

**Conclusion:**

Metabolomics can serve as a holistic tool to identify bottlenecks in metabolic pathways by a non-rational design. Genetic manipulation to release these restrictions could increase the production of succinate.

**Supplementary Information:**

The online version contains supplementary material available at 10.1007/s11306-022-01912-9.

## Introduction

The biological synthesis of high value chemicals has recently attracted lots of interests, as new feedstocks become available and in order to reduce the environmental impact of petrochemical derivatives production. Thus, the United States Department of Energy has declared 12 compounds as building blocks, including the C4-dicarboxylic acids such as succinic, malic and fumaric acids. For instance, the succinic acid market is approximately 20,000–30,000 tons a year, being used principally in four industrial sectors that require high cost raw materials including: (1) detergents and surfactants, (2) ion chelators, (3) food (acidulants and antimicrobial) and (4) pharmaceutical industry (Ahn et al., 2016; Beauprez et al., 2010; Song & Lee, 2006; Zhu et al., 2014). These metabolites can be produced by biological means (Werpy & Petersen, 2004). Succinic acid can be produced through microbial fermentation processes with glycerol which is an interesting and relatively cheap alternative substrate since it is produced as waste from the biodiesel industry and also it has a high carbon content (58.4%) and reduction power (da Silva et al., 2009; Gholami et al., 2014; Samul et al., 2014). Glycerol can be metabolized by several bacterial species (Agarwal et al., 2007; Barros et al., 2013; Lee et al., 2010; Nikel et al., 2008; Scholten et al., 2009) Among them, *Escherichia coli* is an interesting option, because it is one of the best characterized microorganisms for biotechnological applications. Indeed, this bacterium, consumes glycerol and produces succinic acid under anaerobic conditions, when the Tricarboxylic Acid (TCA) cycle splits in two linear pathways, one of which has succinic acid as an end product that is exported out of the cell. For this reason, most of the work related to succinic acid production in *E. coli*, has been carried out under anaerobic conditions. This production has been implemented by using diverse metabolic engineering strategies such as inactivation of fermentative pathways of co-products (lactate, acetate, formate and ethanol) as well as the overexpression of anaplerotic and cataplerotic enzymes. Using these strategies it is possible to obtain biotransformation yields of up to 0.80 mol/mol using glycerol as a carbon source (Blankschien et al., 2010; Liang et al., 2011; Zhang et al., 2010). However, the anaerobic production of succinic acid becomes difficult and slow due to the low growth and substrate consumption rate. Nevertheless*, *in silico models show that the aerobic production of succinic acid is more favorable considering that succinic acid production is associated with growth, in which the maximum molar yield of biomass under aerobic conditions is 0.725 mol/mol which is considerably higher respect to anaerobic condition that is 0.187 mol/mol (Liu et al., 2010; Steinsiek et al., 2011).

The main genetic engineering strategies previously tested for succinic acid production under strictly aerobic conditions involved the linearization of the TCA cycle by deletion of succinic acid dehydrogenase subunits A and B (*sdhAB*) in which succinic acid is exported out of the cell. To improve the biotransformation yield, the glyoxylate shunt has also been activated in this genetic background by deletion of the *iclR* gene (isocitrate lyase regulator), a negative regulator of this pathway’s genes (*aceA;* isocitrate lyase and *aceB*; malate synthase A). This activation has been implemented by deletion of the genes for the enzymes of competitive pathways such as pyruvate oxidase (*poxB*), acetate kinase (*ackA*) and phosphate acetyltransferase (*pta*) (Lin et al., 2005a). For instance using the multiple mutant *ΔsdhABΔpoxBΔackA-ptaΔiclR* 0.67 mol succinic acid/mol glycerol was obtained (Lin et al., 2005b). Other strategies using at least one of these gene deletions have been used by Li et al. (2013). Another strategy previously reported consisted on the mutation of gluconate dehydrogenase (*gnd)* gene involved in the reductive pentose phosphate pathway (PPP), which increased the succinic acid production under anaerobic conditions using glucose or glycerol as the carbon source (Mienda et al., 2016; Zhao et al., 2004). Regardless of such successful strategies, in silico analysis suggest that succinic acid production could achieve higher rates in aerobiosis (Chong et al., 2014). It is therefore necessary to optimize succinic acid precursors through metabolic strategies that prevent carbon leakages and maintain energy and redox balance for cell survival and growth (Zhu et al. 2014), although these carbon leakages are difficult to predict due to the complexity of the biochemical pathways. In the past two decades, the application of metabolomics has proven itself as a helpful approach for a better understanding of the metabolic processes in different biological systems (Martien & Amador-Noguez, 2017). The profiling of metabolites in biological systems has been of interest for many years since the work of Williams et al. (1951) as an early demonstration of “metabolic pattern” unique to individuals (Williams et al., 1951). The analytical platforms most commonly used in this field are gas or liquid chromatography–mass spectrometry (GC–MS or LC–MS) and nuclear magnetic resonance (NMR) spectroscopy (Begley et al., 2009). Other hand, spectroscopic methods have also been used in metabolomic platforms; for instance, Fourier transform infra-red (FT-IR) spectroscopy can be applied as an automated and very rapid holistic approach (Winson et al., 1997) providing biomolecular “fingerprints” made up of the vibrational features of microbial cell components (Naumann et al., 1991) and the chemically-based discrimination of intact microbial cells, which may allow for the detection and identification of the most significant groups of biomolecules in a biological system. Such findings may direct the study towards the appropriate omics platforms and analytical methods (Goodacre et al., 1998). In the absence of a universally applicable analytical platform, complementary data can be compiled using several platforms, to allow for better understanding of a biological process and a more informative conclusion to be made (Begley et al., 2009).

Taking into account the robustness and the advantages of metabolomic platforms, the aim of the present work is to perform a metabolomics analysis of three aerobic succinic acid producer strains with different genetic backgrounds: the base mutant (*ΔsdhAΔack-ptaΔpox*) (Lin et al., 2005a) was used to generate two quintuple strains: (i) with an additional mutation of the *iclR* gene (*ΔsdhAΔack-ptaΔpoxΔiclR*) or (ii) the *gnd* gene (*ΔsdhAΔack-ptaΔpoxΔgnd*). The findings of this study provide a better understanding of the metabolic rewiring processes in these mutants, which may assist the metabolic design and engineering strategies for improving succinic acid production.

## Materials and methods

### Bacterial strains and construction of gene knock out mutant strains

The strains, plasmids and primers used for the construction of multiple mutant strains in this study are listed in Table S1, S2 and S3. The preparation of competent cells, construction of DNA fragments and plasmid transformations were performed using standard procedures (Sambrook et al., 1989). The quadruple mutant *ΔsdhAΔack-ptaΔpox::*kan (M4) and the quintuple mutant (M4-*Δgnd* and M4-*ΔiclR*) strains were constructed in our laboratory following the homologous recombination method described by Datsenko and Wanner (2000) as described (Soto-Varela et al., 2021). Additionally, the M4-*Δgnd* and M4-*ΔiclR* were used as parental strains for the construction of the M4-*ΔgndΔmtlD,* M4-*ΔiclRΔmtlD* and M4-*ΔiclRΔotsA* mutant strains using the same methodology. All of the gene knockout strains were verified by PCR using external and Kanamycin internal primers as shown in Table S1.

### Growth conditions for fermentation and analytical techniques

Assays were carried out in batch culture at 37 °C in a rotary shaker at 200 rpm using. The M9 mineral medium composition in 1 L: 9.97 g Na_2_HPO_4_, 3 g KH_2_PO_4_, 1 g NH_4_Cl, 0.5 g NaCl, 2 mL MgSO_4_ 1 M, 0.1 mL CaCl_2_ 1 M, 3 mL trace element solution (2.3 g/L FeCl_3_–6H_2_O, 0.039 g/L CuSO_4_–5H_2_O, 0.049 g/L ZnSO_4_–7H_2_O, 0.32 g/L MnCl_2_–7H_2_O, 0.129 g/L CoCl_2_–6H_2_O, 0.037 g/L (NH_3_)_6_Mo_7_O_24_–4H_2_O and 0.25 g/L H_3_BO_3_) with 11.5 g/L (125 mM) of glycerol and culture conditions were described in (Soto-Varela et al., 2021) with an initial biomass concentration of 0.031 g CDW/L. For metabolomic analysis, the fermentation was performed using M9 medium supplemented with 2 g/L NaHCO_3_ for 72 h and samples were taken at 0, 10, 24, 31, 48, 55 and 72 h, in M4, M4-*Δgnd* and M4-*ΔiclR* strains. The fermentation for validation of metabolomic analysis were performed up to 48 h in M4-*Δgnd*, *ΔgndΔmtlD*, M4-*ΔiclR*, M4-*ΔiclRΔmtlD* and M4-*ΔiclRΔotsA* strains using M9 medium supplemented with 4 g/L NaHCO_3_ after optimization of culture medium by Soto-Varela et al. (2021) for 48 h since inoculation and samples were taken at 0, 10, 24, 31 and 48 h.

Succinic acid, malic acid, acetic acid, and glycerol quantification from extracellular medium were measured from a 0.22 µm filtered culture supernatant by High Performance Liquid Chromatography (HPLC) using a LaChrom Elite (VWR-Hitachi) equipped with an HPX-87H organic acid column (Bio-Rad, Hercules, CA). The compounds were separated by an isocratic method of acid water with 5 mM H_2_SO_4_ as mobile phase at 0.6 mL/min during 30 min and a column temperature of 50 ºC. The biomass was measured by optical density at 570 nm and used to estimate 1 OD_570_ = 0.31 g cell dry weight (CDW)/L; using the equipment and the methods described by Cofré et al. (2012).

### Metabolomics procedure

For FT-IR analysis, collected bacterial biomass was washed using sterile physiological saline solution (0.9% NaCl), to remove any residuals from the culture medium. Three independent samples were normalized according to their OD_600nm_ by adding saline solution. Normalized bacterial slurries were spotted as 20 µL aliquots onto a FT-IR silicon microplate and dried at 55 °C for ~ 30 min. FT-IR spectra were collected between 4000 and 600 cm^−1^ wavenumbers using a Bruker Equinox 55 infrared spectrometer (Bruker Optics Ltd, Coventry, UK), following our previously published methods (Muhamadali et al., 2015a, 2015b). All spectral data were normalized using extended multiplicative signal correction (EMSC) (Martens et al., 2003), followed by replacing the CO_2_ peak (2400–2275 cm^−1^) with a trend. The pre-processed FT-IR spectral data were subjected to principal components analysis (PCA) (Wold 1987), an unsupervised clustering method to reduce the dimensionality of the data, to classify samples based on their vibrational fingerprint patterns.

For GC–MS analysis, three independent samples were collected and quenched at different timepoints by adding 10 mL of cold (− 48 °C) 60% methanol solution to 5 mL of sample, immediately followed by centrifugation at − 9 °C, 5000×*g* for 10 min to remove the supernatant. All quenched biomass samples were stored at − 80 °C until further analysis. Extraction of the internal metabolites were carried out by suspending the quenched biomass in 1 mL of cold (− 48 °C) 80% methanol solution, followed by snap-freezing in liquid nitrogen and thawing on ice. After a total of three freeze-thawing steps, samples were centrifuged at 15,871×*g*, − 9 °C for 5 min. The supernatants (cell extracts) were transferred to new microcentrifuge tubes, and an aliquot (50 µL) from each sample was taken and combined in a new tube to be used as quality control sample (QC). Internal standard (0.2 mg mL^−1^ succinic-*d*_4_ acid, 0.2 mg mL^−1^ glycine-*d*_5_) was added (100 µL) to all samples, followed by drying at 30 °C for 12 h using a speed vacuum concentration (concentrator 5301; Eppendorf, Cambridge, United Kingdom). All samples were derivatized following a two-step; methoxyamination followed by trimethylsilylation as reported in the literature (Wedge et al., 2011). GC–MS analysis was undertaken on an Agilent Technologies 7200 accurate mass Q-TOF mass spectrometer coupled to a 7890B GC and equipped with a PAL RSI 85 autosampler. The sample (1 μL) was injected onto a VF-5 ms column (30 m × 250 μm × 0.25 μm; Agilent Technologies) with an inlet temperature of 280 °C and a split ratio of 100:1. Helium was used as the carrier gas with a flow rate of 1.5 mL/min and a pressure of 16.2 psi. The chromatography was programmed to begin at 70 °C with a hold time of 4 min, followed by an increase to 300 °C at a rate of 14 °C/min and a final hold time of 4 min before returning to 70 °C. The total runtime per analysis was 24.43 min. The MS was equipped with an electron impact ion source using 70 eV ionisation and a fixed emission of 35 μA. The mass spectrum was collected for the range of 50–550 m/z with an acquisition rate of 5 spectra/s and an acquisition time of 200 ms/spectrum. The GC–MS data were firstly converted to mzXML (Pedrioli et al., 2004) format and imported into R (R Core Development Team, 2015) environment. The data were then deconvolved by using eRah package (Domingo-Almenara et al., 2016). A total number of 612 features were detected and after removing features with over 20% RSD in the QCs 447 features remained for statistical analysis. The peak intensities were log_10_-scaled and then subjected to PCA.

### Parameters calculation, statistical analysis and heat map design

The parameters used in this study are calculated as follows:

$$ {\text{Volumetric }}\,{\text{production}}:\, \, \left[ {{\text{succinic}}} \right] \, \,\left( {{\text{mM}}} \right);\, \,\,{\text{Molar }}\,{\text{yield}}:\,\,\,\frac{{\left[ {{\text{succinic}}} \right]}}{{\left[ {{\text{glycerol }}\,\,{\text{consumed}}} \right]}}\,\,\left( {{\text{mol}}/{\text{mol}}} \right) $$

$$ {\text{Specific }}\,\,{\text{production}}:\,\,\frac{{\left[ {{\text{succinic}}} \right]}}{{{\text{g}}\, CDW}}\,\,({\text{mmol}}\, \times {\text{g }}\,{\text{CDW}}^{ - 1} ); $$

The values of each metabolite obtained from metabolite profiling were analyzed statistically and plotted using three replicates. The identified compounds by GC–MS analysis were curated by selecting those metabolites belong to *E. coli* metabolism using the EcoCyc v. 25.1 database (Keseler et al., 2017). The statistics of univariate data were not normal using Shapiro–Wilk test therefore, U Mann–Whitney was used as a non-parametric test to determine the statistically significant differences between M4, M4-*ΔiclR* and M4-*Δgnd* mutants at 24 and 48 h; by using *P* value < 0.05. A heatmap was performed with metabolites involved in nitrogen metabolism obtained from GC–MS data for analysis of nitrogen (N)-metabolic changes in the three mutants at both times with three replicates. For this purpose, heatmap was performed with Heatmapper software available in the web-enabled heat mapping using Spearman rank correlation of distance measurement method and the clustering method of average linkage (Babicki et al., 2016). For testing of statistically significant values of succinic acid, glycerol and growth in the M4-*ΔiclR*, M4-*Δgnd* mutants, as well as their derivative mutant strains, it was performed a Shapiro–Wilk test for Normality, a Levene’s test for analysis of variance (ANOVA) and a Student-*t*-test for comparing the parameter’s average between two groups with *P* value < 0.05 with a *n* = 4–9. All of the statistical analyses were performed using SPSS® Statistics v.24 software (IBM, Armonk, NY, USA).

## Results

As reported in the literature, three mutant strains capable of producing succinic acid under aerobic conditions (Lin et al. 2005b), were constructed: (i) the *ΔsdhAΔacka-ptaΔpox* strain (M4), (ii) the mutant in which the glyoxylate pathway is also activated (M4-*ΔiclR*)*,* and (iii) the M4-*Δgnd* in which the oxidative PPP is blocked. These three mutants were grown up to 72 h and several metabolomic analyses were performed on these mutants in order to find out new metabolic engineering strategies to improve succinic acid production.

### Growth of *E. coli* mutants and extracellular metabolites

**Fig. 1:**
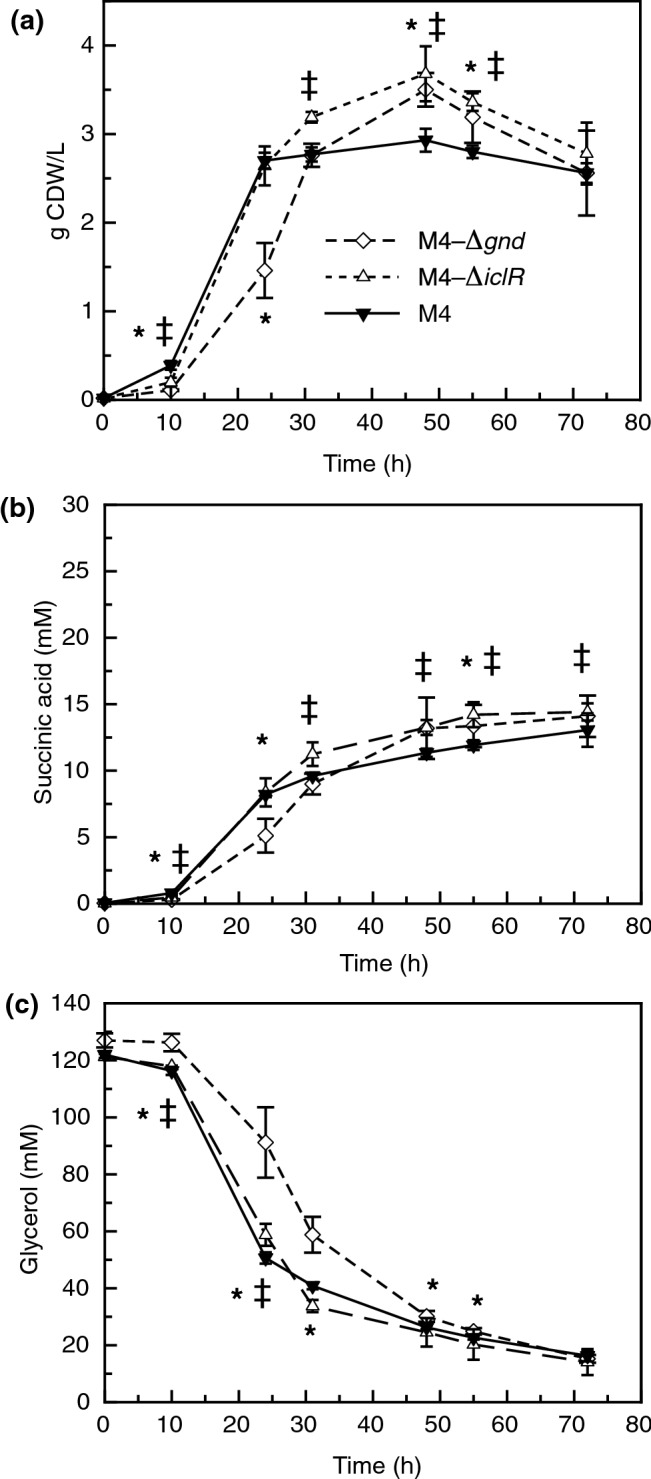
*E. coli* growth profiles (a), succinic production (b) and glycerol in the culture medium (c) of M4 mutant, M4-∆*gnd* and M4-∆*iclR* strains in mineral M9 medium (Soto-Varela, 2021) with glycerol and 2 g/L of bicarbonate over a 72 h experiment. Data points are average ± standard deviation with *n* = 6–9. The asterisk (*) indicates there is statistically significant differences between M4-∆*gnd* an M4 mutant strains and this symbol (‡) indicates the M4-∆*iclR* values are statistically significant differences respect to M4 mutant values by statistics Student’s *t*-test with *P* value < 0.05

Characterization of the growth profiles, extracellular succinic acid, and glycerol concentrations detected in the culture medium, for all three strains (M4, M4-*ΔiclR* and M4-*Δgnd*) are provided in Fig. 1. The growth profiles of the three mutants (Fig. 1a), displayed a higher final biomass yield for the M4-Δ*iclR* achieving a maximum of 3.68 g CDW/L and 3.5 g CDW/L in M4-*Δgnd* at 48 h that were statistically significantly higher than the M4 values. Regarding the succinic acid level (Fig. 1b), the M4 produced less succinic acid (13 mM) than the M4-*Δgnd* and M4-*ΔiclR* mutants (~ 15 mM at 55 h) with statistically significantly differences respect to M4 mutant. It is remarkable that M4-*ΔiclR* has a higher succinic acid production rate with two slopes in log and stationary phases, compared to those of M4 and M4-*Δgnd*. Regarding the glycerol levels in the medium (Fig. 1c), the M4-*ΔiclR* mutant strain consumed 95% of glycerol with higher consumption rate, while the M4 and M4-*Δgnd* mutants consumed around 88%.

### Metabolic profile by GC–MS and Fourier transform infra-red (FT-IR)

**Fig. 2:**
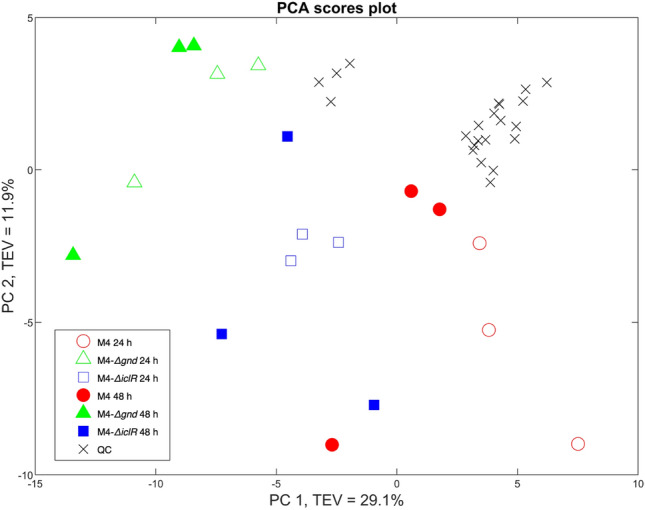
PCA scores plot of the GC–MS data (relative peak areas) from M4 mutant, M4-∆*gnd* and M4-∆*iclR* strains at 24 and 48 h after inoculation (*n* = 3). For plotting PC1 (x-axis) and PC2 (y-axis) were used with of 29.1% and 11.9 of total explained variance (TEV) respectively. QC means quality control samples as described in material and methods section

GC–MS analyses of the samples were conducted to disclose changes in metabolic profiles and principal component analysis (PCA) scores plot of the three mutant strains were obtained (Fig. 2) and the PCA loadings plots (Fig. S1). It is worth noting that the quality control samples (QC) are forming a tight cluster in the PCA score plot (Fig. 2), validating the technical reproducibility of the process. According to the PC1 and PC2 axis there are no significant differences between the time points and the variations observed between the strains that could be explained by a combination of PC1 and PC2. In PC1 axis the M4-*Δgnd* mutant clearly separated from M4-*ΔiclR* and M4 mutants with a total explained variance (TEV) of 29.1%. PC1 *vs* PC2 loadings plot of the GC–MS data was plotted to identify the most significant metabolites contributing to the observed clustering pattern (Supplementary Information Fig. S1, Table S4). The most significant metabolites identified were fructose and glucose (non-phosphorylated) in positive side of PC1 axis (Fig. 2), where M4 mutant is situated, and in contrast, urea, serine and mannitol, were identified as significant on the negative side of PC1 axis, where the M4-*Δgnd* and M4-*ΔiclR* samples are situated. As a complementary analysis, FT-IR was mainly employed as a holistic phenotypic or metabolic fingerprinting approach to provide a general overview of the main changes occurring in different groups of biomolecules and biochemical changes between the investigated strains using PCA. PCA scores plot of all samples (Supplementary Information Fig. 2a), clearly displays the separation of M4-*ΔiclR* at 24 h from both M4 and M4-*Δgnd* strains according to the PC1 axis with TEV of 85.5%. However, it also allowed for the separation of all 24 h samples from those collected after 48 h of incubation. According to the vibrational bands that correspond to the functional groups (Supplementary Information Fig. S2b), the M4-*ΔiclR* strain clearly displayed higher intensities for amide I and II, which are found for instance predominantly in proteins, so the differences of relative abundance could explain the wide separation between the M4-*ΔiclR* at 24 h compared to the rest of the samples (Supplementary Information Fig. S2c). Although with this technique it can also observed a clear separation of M4-*ΔiclR* strain, this methodology cannot provide a metabolite-specific level of identification in such a complex matrix. However, it could be a useful approach to identify a metabolic pattern for screening, in this case, succinic acid-producer strains.

### Analysis of central carbon pathways by carbon flux diagram and heatmap

**Fig. 3:**
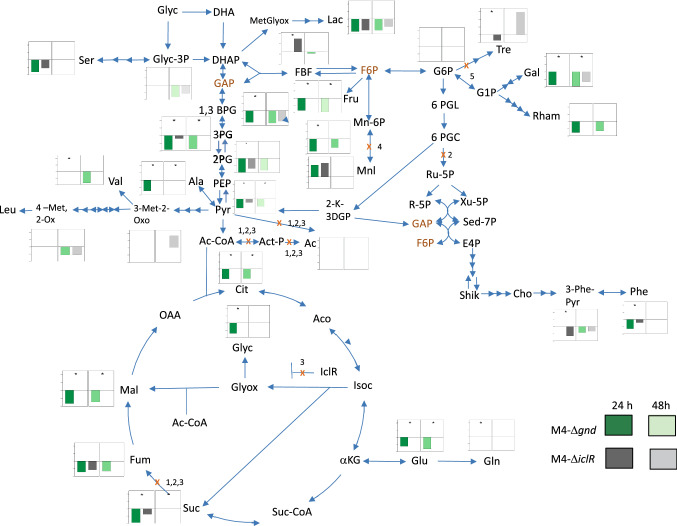
Map of central carbon pathway of glycolysis, TCA cycle, gluconeogenesis, glycerol assimilation, Pentose Phosphate Pathway (PPP), and several aminoacids pathways, was constructed following EcoCyc Database information including plots with the relativized metabolite values of M4∆*gnd* and M4-∆*iclR* strains respect to the M4 values at 24 and 48 h after inoculation with three replicates. In green bars are represented the M4-∆*gnd* respect to M4 values at 24 h (dark green bars) and at 48 h (light green bars). In grey bars are represented the M4-∆*iclR* respect to M4 values at 24 h (dark grey bars) and at 48 h (light grey bars) (*n* = 3). Absence of bars in the graph indicates that there are non-statistically significant differences between the M4-∆*gnd* and M4-∆*iclR* mutants respect to that obtained in M4 strain. The asterisks (*) indicate there is statistically significant differences between M4-∆*gnd* an M4-∆*iclR.* The red cross (X) indicates the blocking of the pathways by the gene deletion in M4 (1), M4-∆*gnd* (2), M4-∆*iclR* (3), M4-∆*gnd*∆*mtlD* and M4-∆*iclR*∆*mtlD* (4) and M4-∆*iclR*∆*otsA* (5) mutant strains*.* The abbreviations for the intermediate metabolites used in this figure are described as follows: Glyc, glycerol; DHA, dihydroxyacetone; Glyc-3P, glycerol 3-Phosphate; DHAP, dihydroxyacetone phosphate; GAP, glyceraldehyde 3-phosphate; 1,3 BPG, 1,3-bisphosphoglycerate; 3PG, 3-phospho-D-glycerate; 2PG, 2-phosphoglycerate; PEP, phosphoenolpyruvate; Pyr, pyruvate; Ac-CoA, acetyl CoA; Cit, citrate; Aco, aconitate; Isoc, isocitrate; αKG, α-ketoglutarate or 2-oxoglutarate; Suc-CoA, succinyl-CoA; Suc, succinate; Fum, fumarate; Mal, malate; OAA, oxaloacetate; Glyox, glyoxylate; Glyc, glycolate; IclR, isocitrate lyase repressor protein; Glu, glutamate; Gln, glutamine; Act-P, Acetyl phosphate; Ac, acetate; Ala, alanine; 3-Met-2-Ox, 3-methyl-2-oxobutanoate; 4-Met,2-Ox, 4-methyl-2-oxopentanoate; Val, valine; Leu, leucine; Ser, serine; MetGlyox, methylglyoxal; Lac, lactate; FBF, fructose 1,6-bisphosphate; F6P, fructose 6-phosphate; G6P, glucose 6-P; Tre, trehalose; G1P, glucose 1-phosphate; Gal, galactose; Rham, rhamnose; Mnl, mannitol; Man-6P, mannose 6-phosphate; Fru, fructose; 6-PGL, 6-phosphoglucono-1,5-lactone; 6PGC, gluconate 6-phosphate; Ru-5P, ribulose 5-phosphate; Xu-5P, xylulose 5-phosphate; R-5P, ribose 5-phosphate; Sed-7P, sedoheptulose 7-phosphate; E4P, erythrose 4-phophate; Shik, shikimate; Cho, chorismate; 3-Phe-Pyr, 3-Phenylpyruvic acid; Phe, phenylalanine; 2K-3DGP, 2-keto-3-deoxy-6-phospho gluconate. In red are indicated the metabolites that are shown twice in the diagram

Although three different groups can be distinguished by PCA, it is worth exploring significant metabolite changes of the identified metabolites. Analysis of the 612 peaks in total that were detected by GC–MS analysis, and 113 of them were positively identified (Table S4) using two orthogonal properties as described by Sumner et al. (2007) and refined further to 63 metabolites (removing redundant compounds and non-identified metabolites). From this list, 48 of them are related to *E. coli* metabolism, which were used for different analysis (Table S5 and S6). In order to investigate the metabolic effects of *gnd* or *iclR* deletion genes in the M4 mutant, the relative peak areas of all the detected metabolites associated with the central carbon metabolism were selected and compared. The relative peak areas of those identified as significant metabolites in the quintuple M4-*ΔiclR* and M4-*Δgnd* mutants with respect to those of the M4 mutant (Table S5) were compared and plotted onto the metabolic map of *E. coli* (Fig. 3). Most of the metabolites, including fumarate and succinate, were detected at lower levels in both quintuple mutants compared to the M4 mutant at 24 h. For most of the glycolysis metabolites the same trend was also found, with statistically significant differences at 24 h in both quintuple mutants respect to the M4. However, fructose 1,6-biphosphate (FBF) at 24 h, threonine, and 3-Methyl-2-Oxobutanoate (3-Met-2-Ox) at 48 h in the M4-*ΔiclR* mutant and mannitol in both mutants at 24 h, displayed higher levels in respect to those obtained in the M4 mutant (Fig. 3). The relative levels of glycerol 3-phosphate (glycerol 3-P) at 24 h were comparable in both mutants but were statistically significantly decreased at 48 h. Differences were also detected in the levels of mannose 6-P, galactose, and rhamnose which were lower in M4-*Δgnd* compared to M4 at both time-points. However, the 1,3 biphosphoglycerate (1,3-BPG) value was higher in M4-*Δgnd* than that of the M4 at 24 h. This value was also higher in the M4-*ΔiclR* than that of the M4 at both times. Interestingly, the M4-*ΔiclR* strain displayed lower trehalose at 24 h, while showing a significant increase at 48 h.

**Fig. 4:**
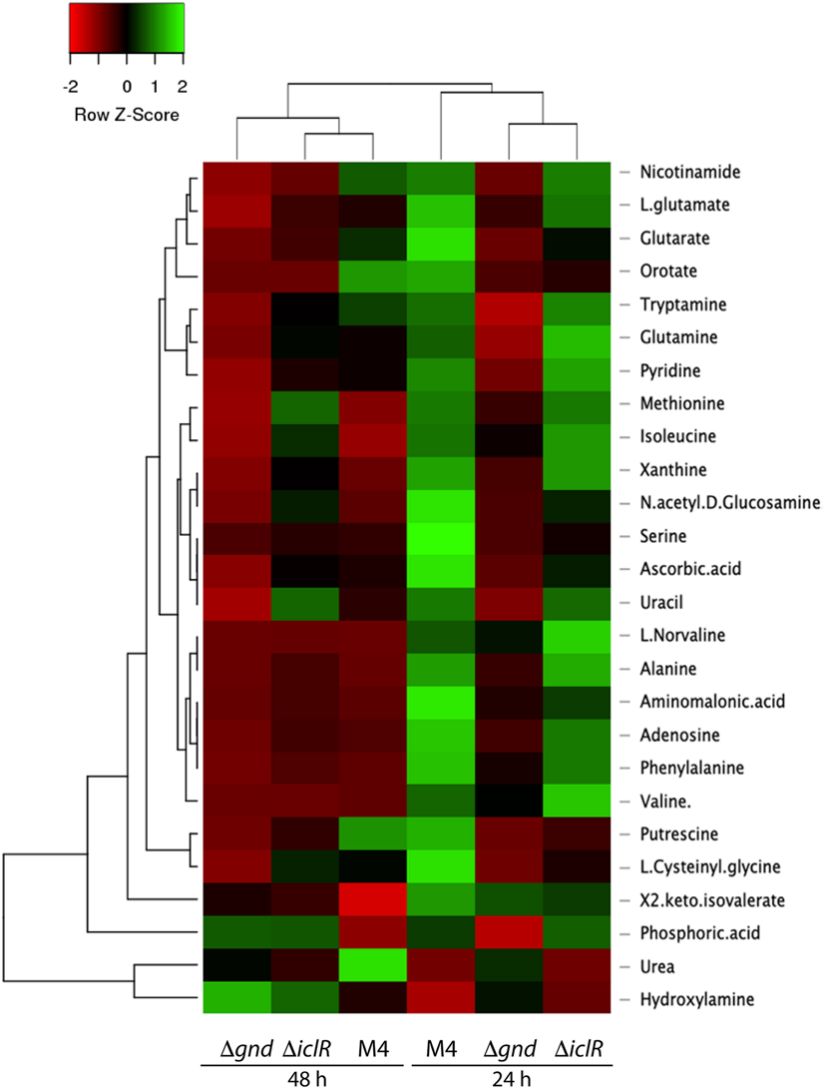
Heatmap performed with metabolites signals average data with *n* = 3 obtained from GC–MS analysis which are involved N-metabolism of M4-∆*gnd* and M4-∆*iclR* strains at 24 and 48 h after inoculation. The software used was Heatmapper with Spearman rank correlation of distance measurement method and the clustering method of average linkage

In order to complement the information of nitrogen metabolism, a heatmap of the relative metabolite abundances by cluster analysis was generated (Fig. 4, Table S6). In the metabolite based dendrogram, two main clusters can be observed, with hydroxylamine and urea being very different compared to the rest of the detected metabolites: urea is higher in M4 and the hydroxylamine in M4-*Δgnd* at 48 h. The second main cluster, is subdivided in two sub-clusters, (i) the phosphoric acid and (ii) cluster is also subdivided into several subclusters whose exact patterns are difficult to interpret. In general terms, no clear metabolic patterns were found, however M4 profiles shows more differences at 24 h, and M4-∆*gnd* shows more differences at 48 h respect to the other (Fig. 4).

### Validation of metabolomic analysis by knock-out of *mtlD* and *otsA *genes in M4-*Δgnd* and M4-*ΔiclR* mutants

Based on the metabolomic analysis described in the previous section, and intending to corroborate the findings of potential metabolic targets to redirect carbon flux towards succinic acid production, the accumulation of mannitol-6P and trehalose could be due to putative C-leakage, so this work suggests removal of the synthesis of both compounds. Mannitol is generated from fructose-6P by two reactions: (1) by mannitol dehydrogenase (MtlD) [1.1.1.17] to produce D-mannitol-1P, and (2) by several phosphatase and/or hydrolase reversible enzymes [3.1.3.22] as well as a predicted transporter [2.7.1.197] (Supplementary Information Fig. S3a). The mannitol dehydrogenase enzyme is codified only by the gene *mtlD* but the second reaction can be performed by eight redundant phosphatase/hydrolase enzymes described so far (Supplementary Information Table S7). This route is the only reaction in *E. coli* for the synthesis of mannitol because this microorganism does not have the enzyme for the synthesis from fructose (Supplementary Information Fig. S3a). To find out if *mtlD* gene has homologous genes *in E. coli,* a sequence analysis by ClustalW was carried out to compare *mtlD* sequence with those of putative hydrolase/phosphatase enzymes genes. This analysis shows a high distance (low similarity) between *mtlD* and the other enzymes’ genes (Supplementary Information, Fig. S4a). This analysis indicates that MtlD function is not redundant in this *E. coli*’s genome. In the case of trehalose, the first reaction from UDP-glucose to trehalose-6P is catalyzed by the trehalose 6P synthase (OtsA) [2.4.1.15], and the second step (trehalose 6-P → trehalose) is catalyzed by trehalose-6P-phosphatase (OtsB) [3.1.3.12]. There is an additional reaction to synthesize trehalose by the phosphotransferase system (PTS) which transport extracellular trehalose by phosphorylation (Crr, TreB) [2.7.1.201] (Supplementary Information Fig. S3b). In order find out a possible redundancy of OtsA activity, a ClustalW analysis of *otsA* and *otsB* genes were performed that revealed only 43.7% of similarity. Additionally BLAST analysis of MtlD and OtsA amino acids sequences, show homology with analogous sequences from other bacteria and no entries of other enzymes have been found with different catalytic activities (Supplementary Information Fig. S4b and c).

**Fig. 5:**
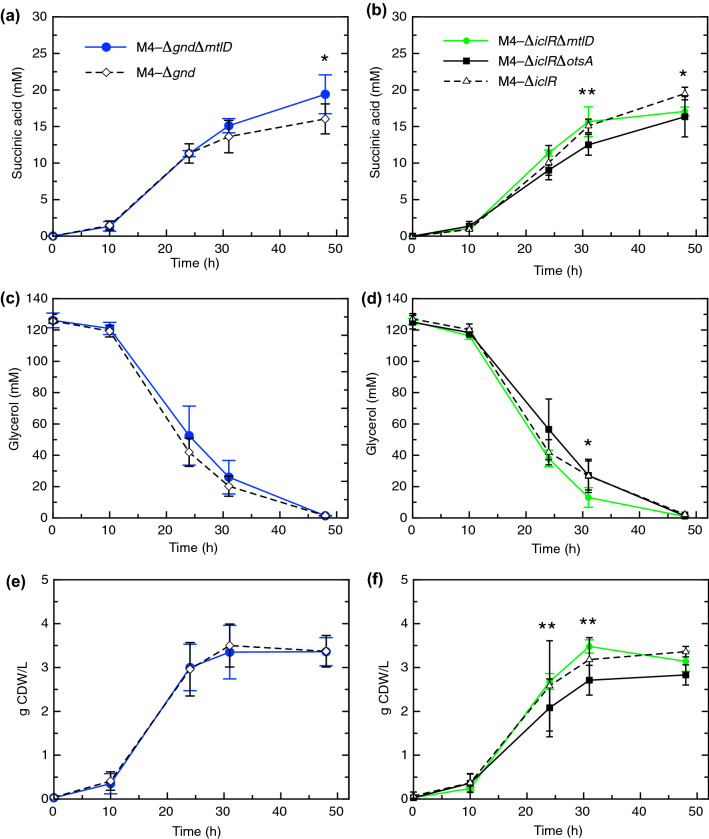
*E. coli* profiles of succinic acid (**a** and **b**), remaining glycerol in the culture medium (**c** and **d**) and g Cell Dried Weight (CDW)/L (**e** and **f**) of: M4-∆*gnd* and M4-∆*gnd*∆*mtlD* mutant strains (**a**, **c** and **e**); M4-∆*iclR*, M4-∆*iclR*∆*mtlD* and M4-∆*iclR*∆*otsA* mutant strains (**b**, **d** and **e**). All of the strains were cultured in M9 medium with glycerol and 4 g/L of bicarbonate over a 48 h experiment. Data points are average ± standard deviation with *n* = 4–9. Asterisks denote statistically significant differences between the group’s averages using Student’s *t*- test with *P* value < 0.05; the comparison of M4-∆*gnd vs* M4-∆*gnd*∆*mtlD* mutant strains (**a**) and M4-∆*iclR vs* M4-∆*iclR*∆*mtlD* (**b**) (*); and the comparison of M4-∆*iclR vs* M4-∆*iclR*∆*otsA* (**b** and **f**) (**). The ANOVA showed that the variances between the groups were homogenous using Levene’s test with *P* value < 0.05

Then the *mtlD* and *otsA* genes that codify mannitol-1-phosphate 5-dehydrogenase and trehalose-6-P synthase enzymes respectively were the candidates to be knocked out because it is assumed that their deletion would avoid the synthesis of Mannitol and trehalose respectively. For this, we firstly knocked out *mtlD* gene, to avoid the mannitol synthesis in both M4-*Δgnd* and M4-*ΔiclR* mutants and secondly we knocked out the *otsA* gene in M4-*ΔiclR* mutant to avoid trehalose accumulation as shown in Fig. 3. The constructed strains, described in Supplementary Information Table S1, were grown with M9 medium supplemented, in this case, with 4 g/L bicarbonate. On the one hand, succinic acid production (mM) in M4-*ΔgndΔmtlD*, at 10 and 24 h time points were not statistically significant different respect to those obtained in the reference strain (M4-*Δgnd*). However, succinic acid production increased from 31 to 48 h, reaching 19.4 mM at this latest time point, which represents a statistically significant 20% higher production than the reference strain (Fig. 5a). In contrast, the growth and the values of the remaining glycerol in the medium for the M4-*ΔgndΔmtlD* strain were not statistically significant different respect to the reference (Fig. 5c and e). On the other hand, the succinic acid production in M4-*ΔiclR* mutant did not improve when *mtlD* and *otsA* genes were deleted, and the deletion of *otsA* (M4-*ΔiclRΔotsA* mutant) even decreased the production of succinic acid (Fig. 5b); as well as the growth respect to M4-*ΔiclR* mutant (Fig. 5f). Nevertheless, the consumption rate of glycerol in M4-*ΔiclRΔmtlD* mutant, increased from 24 up to 48 h and this value showed a statistically significant 11% increase respect to the reference at 31 h (Fig. 5d). In this section, pH was also monitored in these strains and no significant differences were observed, although the pH value in the M4-*ΔgndΔmtlD* strain was slightly lower respect to the reference strain (Supplementary Information Fig. S5a and b). Despite the M4-*Δgnd* and M4-*ΔiclR* strains were defective in acetate biosynthesis genes (*Δack-ptA*, *Δpox*), some acetate leakage was observed with extracellular values in the parental strain identical to those obtained of the subsequently constructed strains (M4-*ΔgndΔmtlD*, M4- *ΔiclRΔmtlD*, M4-*ΔiclRΔotsA*) (Supplementary Information Fig. S5c and d). Therefore, the slightly drop of pH (0.1 value) in M4-*ΔgndΔmtlD* can be exclusively explained by succinic acid production because (1) acetate leakage does not change and (2) the extracellular malate concentration was negligible (< 1 mM) in all of the strains tested in this work (data not shown).

## Discussion

This study aim was to use metabolomics tools to identify key metabolites as targets for potential genetic modifications of *E. coli* strains to improve succinic acid production under aerobic conditions. To this end, three mutant strains, previously described as capable of producing succinic acid under aerobic conditions (Lin et al. 2005b), were constructed: M4, M4-*ΔiclR*, M4-*Δgnd* mutant strains (Soto-Varela et al., 2021). Comparative analysis of the metabolome using complementary techniques such as FT-IR spectroscopy, GC–MS and targeted HPLC, revealed interesting differences in the behavior and phenotypic profiles of these three mutants. The FT-IR analysis indicated that the metabolic fingerprint of M4-*ΔiclR* was significantly different from the rest of the samples from M4 and M4-*Δgnd* strains (Supplementary Information Fig. 2a and c). This distinctive profile is consistent with the differences in the growth from 31 up to 55 h (Fig. 1a) and the growth rate (Table 1), which were higher respect to the M4 mutant, and the glycerol consumption rate (since 10 up to 31 h) that was also higher (Fig. 1c). However, after the first 31 h, a decrease of glycerol consumption rate was observed in all three strains, which agree with (Lin et al. 2005b) findings, who reported a decrease in the carbon source consumption (glucose in that case) after 24 h. For this reason, a strategy to increase growth and productivity could be focused in improving glycerol assimilation in both quintuple mutants after 24 h, taking into account what was described by Jiang et al. (2016). Our results also confirm that glycerol is a more favorable carbon source than glucose for the M4 genetic background, since feeding with glucose (10 g/L [55.51 mM]) produced 4 mM of succinic acid (Lin et al., 2005a) resulting in 3.5% of theoretical molar yield, while the same strain produces 8 mM after 24 h using glycerol (11.5 g/L [125 mM]) as this work describes, which is 6% of theoretical molar yield. This evidence is in agreement with the GC–MS analysis, which indicates that glycerol 3-P values in both quintuple mutants had decreased compared to the M4 strain values. On the other hand, the mutation of *iclR* gene in the M4 mutant slightly increased succinic acid production at 48 h (Fig. 1b) using glycerol and in a lesser extent to that obtained using glucose (Lin et al. 2005b). Additionally, in this study, a similar slight increase in extracellular succinic acid was observed in the M4-*Δgnd* strain. The finding of differences in the relative concentration of these metabolites may also suggest the presence of key regulation points, and possible competitive metabolites and pathways that can be considered carbon leakages. The GC–MS results also implied that carbon metabolism seems to be more active in the M4 strain. In fact, the differences detected between the strains by PCA could be explained mainly by changes in various sugars levels such as fructose and glucose in the M4 mutant. These results are also agree with the FT-IR findings, which suggested major changes in polysaccharide vibrational regions (900–1200 cm^−1^), as well as changes in the protein and peptide levels which could also be linked to the expression of different enzymes, contributing to the upregulation of various pathways. The PCA shows how nitrogen metabolism is more active in M4 and M4-*ΔiclR* mutant, not only in terms of amino acids metabolism, but also in precursors of nitrogenous bases such as uracil and xanthine (Fig. 2, Fig. 4 and Table S4), reflecting the higher growth rate of both strains respect to M4-*Δgnd* (Table 1). Nitrogen catabolism at 48 h seems to be also more active, which may explain the diauxic shift in the growth curves (Fig. 1a) as products such as putrescine and urea were detected in M4. Remarkably, urea and serine were key metabolites for separation between M4-*Δgnd*, M4-*ΔiclR* and M4 samples in PCA (Fig. 2, Table S4) and the separation of M4-*ΔiclR* is also shown by FT-IR analysis that can explain by the possible abundance, in agreement to amine or amides (C = O, N–H, C-N) functional groups (Supplementary Information Fig. S2) of asparagine but not in glutamine because similar level of glutamine was detected in the three mutants at the two time points (Fig. 3). Additional analysis by heatmap performed with 26 identified compounds related to N metabolism (Fig. 4 and Table S6), and a metabolite-based dendrogram showed two main clusters. The dendrogram revealed that relative quantification of hydroxylamine and urea at two time points are very different to the rest of the detected metabolites. Urea is higher in M4 and the hydroxylamine in M4-*Δgnd* at 48 h. Urea is involved in amino acid catabolism as the main nitrogen waste product, whereas hydroxylamine is involved in ammonium generation from a reduced electron acceptor, or can be also generated from glutathione. This effect could probably be due to its important role in the regulation of the nitrogen assimilation. Glutamine synthetase, which catalyze the amination of the glutamate to glutamine is a key enzyme in ammonia assimilation and it has a feedback regulation (Goss et al., 2001; Stadtman, 2001). This enzyme may also be used to control the carbon flux to α-ketoglutarate and therefore to succinic acid. This metabolic redirection may be modulated by addition of different NH_4_ concentrations to the culture medium. In the M4-*Δgnd* strain most of the nitrogen compounds levels were lower than those obtained in M4 and M4-*ΔiclR* strains, probably due to the blocking of the oxidative phase of the PPP (through *gnd* gene deletion) and the theoretical NADPH depletion. This lower NADPH/NADP^+^ balance should affect many anabolic reactions (Giraud & Naismith, 2000; McCourt et al., 2004; Yu et al., 2011) and may be a bottleneck in the metabolism of this mutant. This effect was also reported by Zhao et al. (2004), who mutated the *zwf* gene, that encode the glucose 6-P dehydrogenase, although in this case only acetate was used as carbon source. On the other hand, it seems that a key point affected by *iclR* mutation is the control of the gluconeogenesis and glycolysis pathways, evidenced by the accumulation of fructose-1,6-BP in this strain at 24 h (Fig. 3). Furthermore, glycerol is a substrate that promotes gluconeogenesis due to the need of sugars synthesis (Peng & Shimizu, 2003) since glycerol and bicarbonate are the unique carbon sources. In the M4-*ΔiclR*, carbon leakage is attenuated due to the fact that in this mutant the activation of the glyoxylate shunt lead to a drop of the two decarboxylation steps of the TCA cycle, allowing the formation of metabolic intermediates from Acetyl-CoA without carbon lost (Mainguet et al., 2013). This could explain the increment in the synthesis of sugars like trehalose and mannitol (Fig. 3)*.* Therefore, the regulation of this step, mainly in the M4-*ΔiclR*, is key point to redirect the carbon flux through glycolysis to the TCA cycle to promote the production of metabolites of interest. This unbalance of the central carbon pathways leads to an increment of trehalose in M4-*ΔiclR* at 48 h, probably caused by an up-regulation of the *otsA* and *otsB* genes which encode the trehalose 6-P synthetase and trehalose-6-P phosphatase enzymes, respectively which are responsible for synthesis of high concentrations of internal trehalose during the transition to stationary phase (Hengge-Aronis et al., 1991), or particularly osmotic stress conditions (Giaever et al., 1988). Trehalose could be considered as a carbon leakage, because it is not a product of biotechnological interest, but it has an important role as osmoprotectant compound in physiological stress of the bacteria. For this, the deletion of *otsA* gene in the M4-*ΔiclR* may improve succinic acid synthesis. In the case of mannitol, it is worth noting that there was an increase in production in both quintuple mutants at 24 h (Fig. 3) compared to that of the M4 strain, being this one of the metabolites that explains the differences between the three strains in the PCA (Fig. 2). In order to validate these hypotheses, the deletion of *otsA* gene on M4-*ΔiclR* and the deletion of *mtlD* gene on both quintuple mutants were carried out. The *mtlD* deletion in M4-*Δgnd* increased succinic acid production around 20% respect to the reference at 48 h (Fig. 5a) and specific production (Table 1) at both time points, without altering the growth curve along 48 h, growth rate of log phase (Fig. 5b and Table 1) and glycerol consumption (Fig. 5c). Mannitol is the most common natural hexitol and can be used as a carbon source in *E. coli* in a reaction catalyzed by MtlD enzyme, which is involved in the reversible conversion of: D-mannitol 1-P + NAD^+^ ←→ Fructose 6-P + NADH + H^+^ (Reshamwala et al., 2014). Therefore, the blockage of mannitol synthesis in gluconeogenesis may avoid C leakage and increase C-flux towards succinic acid. We found that, although the deletion of *mtlD* on the M4-*ΔiclR* mutant, does not have a significant effect on succinic acid production, glycerol consumption rate increased at 31 h, so this increment of C-flux is probably diverted to growth but not to succinic acid net production because no differences were observed respect to the reference strain (Fig. 5b, d and f). On the other hand, the other possible C leakage identified in this study is the synthesis and accumulation of trehalose, a disaccharide which is synthesized from Glucose and Glucose 6-P by trehalose 6-P synthase (OtsA) enzyme under conditions of high osmolarity (Giaever et al., 1988). Although trehalose accumulation is observed in the M4-*ΔiclR* mutant at 48 h (Fig. 3), the deletion of *otsA* enzyme gene, not only did not enhance succinic acid production but also diminish this production as well as the glycerol consumption and affected to the growth curve (Fig. 5b, d and f) and the growth rate (Table 1). As described above, endogenous trehalose is synthesized in stationary phase under physiological stress conditions. As described above, the deletion of *otsA* gene affects significantly to the growth and consequently to succinic acid production. It has been reported before that *otsA* mutants are viable but osmotically sensitive in minimal medium (Giaever et al., 1988), which is the same medium used in this work. The M4-*ΔiclRΔotsA* mutant strain is probably also more sensitive to extracellular succinic acid, and this idea is supported by the fact that overexpression of *otsA* gene improves ethanol tolerance in an engineered strain (Woodruff et al., 2013). Therefore, the hypothesis of blocking trehalose synthesis as a C-leakage to increase C-flux towards succinic acid is not supported by the results obtained in the M4-*ΔiclRΔotsA* mutant since trehalose is performing a significant role in the tolerance to succinic acid. In this sense, it is important to highlight how metabolomics can evidence new targets or phenotypes that the rational metabolic engineering design cannot predict, contributing therefore the implementation of new enhanced producer strains.

**Table 1:** Comparison of the parameters of: growth rate, molar yields, and specific succinic acid production in M4, M4-∆*gnd*, M4-∆*iclR,* M4-∆*gnd*∆*mtlD* (in bold), M4-∆*iclR*∆*mtlD*, and M4-∆*iclR*∆*otsA* mutant strains*.* Means and standard deviations were calculated using between four and nine replicates

Bicarbonate in M9 medium (HNaCO_3_)	Mutant strains	Growth rate (µ)	Molar yield (mol succinic acid /mol glycerol consumed)		Specific succinic acid production (mmol × g CDW^−1^)	
			24 h	48 h	24 h	48 h
2 g/L	M4	0.12 ± 0.01	0.12 ± 0.00	0.12 ± 0.01	3.05 ± 0.10	3.89 ± 0.24
	M4-∆*gnd*	0.13 ± 0.01	0.14 ± 0.01	0.14 ± 0.02	3.48 ± 0.23	3.80 ± 0.81
	M4-∆*iclR*	0.14 ± 0.01	0.13 ± 0.01	0.14 ± 0.01	3.16 ± 0.18	3.72 ± 0.18
4 g/L	M4-∆*gnd*	0.15 ± 0.02	0.14 ± 0.03	0.13 ± 0.02	4.03 ± 1.23	4.71 ± 1.07
	M4-∆*gnd*∆*mtlD*	0.15 ± 0.03	**0.15 ± 0.03**	**0.16 ± 0.02**	**4.55 ± 1.65**	**5.93 ± 1.20**
	M4-∆*iclR*	0.14 ± 0.02	0.15 ± 0.03	0.15 ± 0.01	5.69 ± 1.65	5.94 ± 0.20
	M4-∆*iclR*∆*mtlD*	0.16 ± 0.01	0.13 ± 0.01	0.14 ± 0.01	4.26 ± 0.46	5.45 ± 0.43
	M4-∆*iclR*∆*otsA*	0.11 ± 0.02	0.14 ± 0.05	0.13 ± 0.03	4.75 ± 1.55	5.88 ± 1.48

## Conclusions

The results obtained in this study evidence that the deletion of mannitol synthesis is a successful metabolic engineering strategy to improve succinic acid production of the M4-*Δgnd* mutant, although nitrogen metabolism was depleted as observed with FT-IR and GC–MS. However, the depletion of trehalose synthesis did not have a synergistic effect using M4-*ΔiclR* as genetic background, which evidence the complexity of the metabolic pathways and regulatory networks and their application to metabolic engineering.

## Supplementary Information

Below is the link to the electronic supplementary material.

Supplementary file1 (DOCX 1028 kb)

## Data Availability

The metabolomics datasets analyzed during the current study, which have been summarized in Supplementary Information Table S4, S5 and S6, are available at the NIH Common Fund’s National Metabolomics Data Repository (NMDR) website, the Metabolomics Workbench, https://www.metabolomicsworkbench.org where it has been assigned Project ID PR000898. The data can be accessed directly via it’s Project https://doi.org/10.21228/M8MD6W.
