# Supplementary material for: Metabolomics for the design of new metabolic engineering strategies for improving aerobic succinic acid production in Escherichia coli
Source: Metabolomics. 2022 Jul 20;18(8):56. doi: 10.1007/s11306-022-01912-9 (PMC9300530; doi:10.1007/s11306-022-01912-9)
Supplement: Supplementary file 1 — Supplementary file1 (DOCX 1028 kb) [file 11306_2022_1912_MOESM1_ESM.docx]

**SUPPLEMENTARY MATERIAL**

**Metabolomics for the design of new metabolic engineering strategies for improving aerobic succinic acid production in *Escherichia coli***

Antonio Valle^1†*^, Zamira E. Soto-Varela^2,3^, Howbeer Muhamadali^4,5^, Katherine A. Hollywood^6^, Yun Xu^4,5^, Jonathan R. Lloyd^7^, Royston Goodacre^4,5^, Domingo Cantero^2†^, Gema Cabrera^2†^ and Jorge Bolivar^1‡*^

^1^ Department of Biomedicine, Biotechnology and Public Health-Biochemistry and Molecular Biology, Campus Universitario de Puerto Real, University of Cadiz, 11510, Puerto Real, Cadiz, Spain.

^2^ Department of Chemical Engineering and Food Technology, Campus Universitario de Puerto Real, University of Cadiz, 11510 Puerto Real, Cadiz, Spain.

^3^ Faculty of Basic and Biomedical Sciences, Universidad Simón Bolívar, 080020. Barranquilla, Colombia

^4^ School of Chemistry, Manchester Institute of Biotechnology, University of Manchester, Manchester, M1 7DN, UK.

^5^ Department of Biochemistry and Systems Biology, Institute of Integrative Systems, Molecular and Integrative Biology, University of Liverpool, Biosciences Building, Crown Street, Liverpool L69 7ZB, UK

^6^ Manchester Centre for Synthetic Biology of Fine and Speciality Chemicals (SYNBIOCHEM), Manchester Institute of Biotechnology, The University of Manchester, Manchester M1 7DN, UK

^7^ Williamson Research Centre, School of Earth & Environmental Sciences, University of Manchester, Manchester, M13 9PL, UK.

^†^ Institute of Viticulture and Agri-Food Research (IVAGRO) - International Campus of Excellence (ceiA3), University of Cadiz.

^‡^ Institute of Biomolecules (INBIO), University of Cadiz.

*Corresponding authors: [antonio.valle@uca.es](mailto:antonio.valle@uca.es) and jorge.bolivar@uca.es. Department of Biomedicine, Biotechnology and Public Health-Biochemistry and Molecular Biology, University of Cadiz, Campus Universitario de Puerto Real, 11510, Puerto Real, Cadiz, Spain. Tel.: +34 956 012045 / +34 956 012791.

**Table S1.** List of the *E. coli* mutant strains used in this study.

| **Name** | **Genotype** | **Source or reference** |
| --- | --- | --- |
| M1 | *E. coli K12* BW25113 *∆sdhA*::Kan | Keio Collection (JW ID: [JW0713-KC](http://ecoli.naist.jp/GB/index.php/search-info?id=23&gbid=JW0713)) |
| M4 | *E. coli K12 ∆sdhA∆ack-pta∆pox*::kan | Soto-Varela (2021) |
| M4-Δ*iclR* | *E. coli K12 ∆sdhA∆ack-pta∆pox∆iclR:*:kan | Soto-Varela (2021) |
| M4-Δ*gnd* | *E. coli K12 ∆sdhA*∆*ack-pta∆pox∆gnd*:kan | Soto-Varela (2021) |
| M4-Δ*gnd*Δ*mtlD* | *E. coli K12 ∆sdhA*∆*ack-pta∆pox∆gnd*Δ*mtlD*:kan | This study |
| M4-Δ*iclR*Δ*mtlD* | *E. coli K12 ∆sdhA*∆*ack-pta∆pox∆iclR*Δ*mtlD*:kan | This study |
| M4-Δ*iclR*Δ*otsA* | *E. coli K12 ∆sdhA*∆*ack-pta∆pox∆iclR*Δ*otsA*:kan | This study |

**Table S2.** List of primers used in this study

| **Primers** | **Sequence 5’ 🡪 3’** | **Source or reference** |
| --- | --- | --- |
| *poxB* H1P4 | GATGAACTAAACTTGTTACCGTTATCACATTCAGGAGATGGAGAACCATGATTCCGGGGATCCGTCGACC | Keio Collection |
| *poxB* H2P1 | CCTTATTATGACGGGAAATGCCACCCTTTTTACCTTAGCCAGTTTGTTTTGTGTAGGCTGGAGCTGCTTC | Keio Collection |
| *ack-pta* H1P4 | TGGCTCCCTGACGTTTTTTTAGCCACGTATCAATTATAGGTACTTCCATGATTCCGGGGATCCGTCGACC | Keio Collection |
| *ack-pta* H2P1 | GCAGCGCAAAGCTGCGGATGATGACGAGATTACTGCTGCTGTGCAGACTGGTGTAGGCTGGAGCTGCTTC | Keio Collection |
| *iclR* H1P4 | CAATAAAAATGAAAATGATTTCCACGATACAGAAAAAAGAGACTGTCATGATTCCGGGGATCCGTCGACC | Keio Collection |
| *iclR* H2P1 | AGAATATTGCCTCTGCCCGCCAGAAAAAGTCAGCGCATTCCACCGTACGCGTGTAGGCTGGAGCTGCTTC | Keio Collection |
| *gnd* H1P4 | AGGCCGCGAGCATTCAGCGCGGTGATCACACCTGACAGGAGTATGTAATGATTCCGGGGATCCGTCGACC | Keio Collection |
| *gnd* H2P1 | AGGCCGCGAGCATTCAGCGCGGTGATCACACCTGACAGGAGTATGTAATGGTGTAGGCTGGAGCTGCTTC | Keio Collection |
| *sdhA*-F | GAACAGCCTATACTGCCGCC | Soto-Varela (2021) |
| *sdhA*-R | TTTATGCTTACTTCGCCGTGG | Soto-Varela (2021) |
| *pox*-F | GGCTGCTGTAAGACAAAAGTGG | Soto-Varela (2021) |
| *pox*-R | TCAAACAGATAGTTATGCGCGG | Soto-Varela (2021) |
| *ack-pta*-F | ATTATCCGGCGTTGACATGC | Soto-Varela (2021) |
| *ack-pta*-R | TGGATCTACGACTTTGCGTG | Soto-Varela (2021) |
| *iclR*-F | TTTGCTGCTCACACTTGCTC | Soto-Varela (2021) |
| *iclR*-R | GGTGTTCATTTGTCTGGGCTG | Soto-Varela (2021) |
| *gnd-*F | TTGTGCGTGTAAATGGCTTCG | Soto-Varela (2021) |
| *gnd-*R | GGATCATAGTCGGTTGGAGTG | Soto-Varela (2021) |
| Kt-R | CGGCCACAGTCGATGAATCC | Datsenko & Wanner (2000) |
| Kt-F | CGGTGCCCTGAATGAACTGC | Datsenko & Wanner (2000) |
| *mtlD* H1P4 | CCTCACCCCAGCCCTCTCGGGTAAAAACATTGATGAAGGTTAATACTATGATTCCGGGGATCCGTCGACC | This study |
| *mtlD* H2P1 | GCGCAGGGTGTCCTGCGCCTGGTCCACCATTATTGCATTGCTTTATAAGCTGTAGGCTGGAGCTGCTTCG | This study |
| *mtlD*-F | TAATCCAATCCCACCCTCTCC | This study |
| *mtlD-R* | GGTTTGTTCCATTGTTGCCTGC |  |
| *otsA* H2P4 | AATGATAACCACCGCATTACAACAAAAAAGAGAAAATAACAGGAGTGATGATTCCGGGGATCCGTCGACC | This study |
| *otsA* H1P1 | TAGTTCCACTTACGGGAGATTAACCGCTCCTACGCAAGCTTTGGAAAGGTTGTAGGCTGGAGCTGCTTCG | This study |
| *otsA* PR | CCGAGTTGGGATTGCGTCG | This study |

**Table S3.** List of plasmids used in this study

| **Plasmids** | **Sites** | **Source** |
| --- | --- | --- |
| pKD13 | oriR6Kγ, tL3LAM(ter) bla (Amp^R^) rgnB(ter) Tn5neomycin P-trans (Kan^R^) | Coli Genetic Stock Center (CGSC). University of Yale |
| pCP20 | cI857λ bla (Amp^R^) cat FLP, repA101 (ts) | CGSC. |
| pKD46 | repA101(ts) P_BAD_ promoter γ−β−exo, oriR101 bla (Amp^R^) | CGSC |

**Table S4. Full name of 113 identified compounds from GC-MS analysis.** In this list it is shown the match factor and the identifier (id). PC1 and PC2 loadings scores of PCA are referred in Fig. 3.

| **Compound name (synonim)** | **Match Factor** | **id** | **PC 1** | **PC 2** |
| --- | --- | --- | --- | --- |
| Acetic acid | 65.66 | 12 | 0.020 | 0.006 |
| Alanyl-alanine | 69.63 | 17 | 0.014 | 0.035 |
| Pyruvate | 71.85 | 48 | 0.055 | 0.033 |
| Propane-1,2-diol | 98.28 | 51 | -0.002 | 0.002 |
| Pyridine | 98.01 | 71 | -0.001 | 0.014 |
| Glucose | 89.83 | 72 | -0.003 | 0.046 |
| Propane-1,3-diol | 87.74 | 78 | 0.002 | 0.007 |
| Lactate | 98.02 | 81 | 0.031 | -0.003 |
| Glycolic acid (glycolate) | 91.00 | 88 | -0.002 | 0.000 |
| Valine | 99.65 | 95 | 0.004 | 0.001 |
| Sarcosine | 99.74 | 99 | 0.001 | 0.002 |
| Keto-isovaleric acid (3-methyl-2-oxobutanoate) | 95.98 | 105 | 0.002 | 0.019 |
| Hydroxylamine | 96.38 | 106 | -0.003 | -0.011 |
| Methionine | 94.37 | 109 | 0.000 | 0.001 |
| Keto-isovaleric acid (3-methyl-2-oxobutanoate) | 88.07 | 114 | 0.000 | 0.003 |
| Pyridine | 96.99 | 118 | 0.008 | 0.010 |
| Butanoic acid | 87.51 | 125 | 0.037 | 0.050 |
| Butanoic acid | 92.14 | 126 | -0.001 | 0.003 |
| Phosphoric acid | 84.09 | 134 | 0.029 | 0.035 |
| Succinic acid | 95.17 | 137 | 0.017 | 0.052 |
| 4-methyl-2-oxopentanoate | 87.32 | 148 | 0.052 | 0.020 |
| L-Norvaline | 99.37 | 149 | 0.013 | 0.003 |
| Tryptamine | 99.37 | 156 | 0.000 | 0.000 |
| Butanoic acid | 84.24 | 158 | -0.010 | -0.012 |
| Serine | 93.25 | 171 | 0.023 | 0.039 |
| Urea | 90.05 | 172 | 0.031 | 0.031 |
| Tryptamine | 99.36 | 176 | 0.002 | 0.003 |
| Phosphoric acid | 87.45 | 177 | -0.002 | 0.033 |
| Phosphoric acid | 91.57 | 179 | -0.008 | -0.014 |
| Isoleucine | 98.49 | 184 | -0.043 | 0.000 |
| Aminomalonic acid | 92.52 | 185 | -0.011 | 0.022 |
| Succinic acid | 92.13 | 195 | 0.000 | 0.008 |
| Glyceric acid | 79.91 | 197 | 0.002 | -0.017 |
| Uracil | 91.66 | 200 | -0.038 | -0.096 |
| Fumaric acid (fumarate) | 95.71 | 203 | -0.002 | -0.004 |
| Serine | 88.46 | 204 | 0.040 | 0.036 |
| L-alanine | 95.86 | 208 | -0.003 | 0.000 |
| L-alanine | 88.70 | 229 | 0.044 | 0.015 |
| Tryptamine | 99.26 | 235 | 0.030 | 0.042 |
| Malic acid (*RS*-malate) | 69.13 | 243 | 0.000 | -0.002 |
| Glutamine | 92.52 | 246 | 0.011 | 0.017 |
| Nicotinamide | 92.36 | 248 | 0.052 | 0.037 |
| Malic acid | 92.99 | 250 | 0.017 | 0.034 |
| Galactose | 90.93 | 256 | 0.009 | 0.001 |
| Nicotinamide | 94.58 | 261 | -0.003 | 0.051 |
| L-methionine | 97.66 | 272 | 0.004 | 0.009 |
| 1-Pyrroline-3-hydroxy-5-carboxylic-acid | 99.83 | 274 | 0.043 | 0.017 |
| Butanoic acid | 96.85 | 275 | 0.045 | 0.016 |
| Glutamic acid | 95.31 | 276 | 0.006 | 0.009 |
| Glutaric acid (glutarate) | 98.95 | 291 | 0.015 | 0.035 |
| Glutaric acid (glutarate) | 88.60 | 292 | 0.017 | -0.008 |
| Galactose | 98.09 | 297 | 0.001 | 0.005 |
| Lactate | 92.46 | 299 | 0.013 | 0.002 |
| 3-Phenylpyruvic acid (3-phenyl-2-oxopropanoate) | 95.83 | 301 | 0.034 | 0.010 |
| Glutamic acid (glutamate) | 95.61 | 307 | 0.048 | -0.005 |
| Ascorbic acid (L-ascorbate) | 86.14 | 308 | 0.046 | 0.010 |
| Phenylalanine | 95.30 | 313 | 0.033 | 0.000 |
| Benzoic acid (benzoate) | 88.70 | 315 | -0.018 | -0.001 |
| Galactose | 96.79 | 317 | 0.018 | 0.019 |
| Pyrophosphate | 96.66 | 320 | -0.002 | 0.141 |
| Glucose | 96.36 | 325 | 0.003 | 0.061 |
| Galactose | 92.99 | 341 | 0.004 | -0.004 |
| Rhamnose | 98.45 | 342 | -0.005 | 0.007 |
| Lactulose | 89.07 | 350 | -0.001 | 0.144 |
| Putrescine | 98.46 | 353 | 0.045 | 0.086 |
| Orotate | 92.11 | 355 | -0.001 | 0.050 |
| Glycerol-3-phosphate | 86.97 | 357 | -0.017 | 0.041 |
| Glycerol-3-phosphate | 78.29 | 359 | -0.016 | -0.016 |
| Glyceric acid-2-phosphate (2-phospho-D-glycerate) | 79.42 | 365 | 0.026 | 0.032 |
| Fructose derivate | 91.99 | 371 | 0.033 | 0.063 |
| Fructose | 90.32 | 372 | 0.045 | 0.075 |
| Glyceric acid-3-phosphate (3-phospho-D-glycerate) | 89.28 | 374 | 0.021 | 0.012 |
| D-fructose | 97.33 | 377 | -0.058 | -0.042 |
| Citric acid | 96.31 | 378 | 0.050 | -0.013 |
| Galactose | 98.37 | 382 | 0.058 | -0.022 |
| Tryptamine | 99.53 | 383 | 0.043 | 0.085 |
| Citric acid (citrate) | 67.85 | 386 | 0.036 | -0.020 |
| Citric acid (citrate) | 79.14 | 387 | 0.035 | -0.020 |
| Galactose | 85.52 | 396 | 0.038 | -0.008 |
| Galactose | 86.79 | 397 | 0.072 | 0.082 |
| Galactose | 86.94 | 401 | 0.006 | -0.019 |
| Glucose | 95.82 | 402 | 0.076 | -0.038 |
| Gluconic acid (D-gluconate) | 81.13 | 403 | 0.049 | -0.032 |
| Galactose | 86.51 | 404 | 0.084 | -0.032 |
| Lactate | 98.75 | 405 | 0.036 | -0.029 |
| Galactose | 84.95 | 406 | 0.025 | 0.004 |
| Galactose | 70.93 | 408 | 0.103 | -0.019 |
| Galactose | 90.19 | 409 | 0.060 | -0.031 |
| Pyruvic acid (=pyruvate) | 96.84 | 410 | -0.006 | 0.002 |
| Galactose | 96.44 | 426 | 0.116 | -0.036 |
| Glutarate | 89.86 | 434 | 0.039 | 0.030 |
| Galactose | 78.97 | 438 | 0.031 | 0.011 |
| Xanthine | 90.04 | 441 | -0.009 | 0.003 |
| Cysteinyl-glycine | 91.03 | 454 | -0.008 | 0.016 |
| N-acetyl-glucosamine | 83.86 | 461 | 0.027 | -0.007 |
| Glyceric acid-1,3-diphosphate (1,3-bisphospho-D-glycerate) | 74.45 | 479 | 0.014 | 0.115 |
| Fructose | 69.68 | 507 | 0.074 | -0.032 |
| Mannitol | 84.80 | 515 | 0.043 | -0.012 |
| Mannitol | 80.27 | 524 | 0.045 | -0.093 |
| Glucose-6-phosphate | 87.83 | 527 | 0.052 | -0.007 |
| Mannose-6-phosphate | 80.88 | 529 | 0.045 | -0.004 |
| Glucose-6-phosphate | 86.84 | 539 | 0.043 | -0.008 |
| Fructose-1,6-diphosphate (β-D-Fructose 1,6 bisphosphate) | 86.23 | 544 | 0.002 | -0.010 |
| Fructose Derivate | 78.91 | 546 | 0.033 | 0.010 |
| Gluconic acid (D-gluconate) | 87.31 | 553 | -0.006 | -0.002 |
| 6-deoxy-mannose | 94.73 | 576 | 0.029 | 0.041 |
| Fructose Derivate | 93.50 | 583 | 0.018 | 0.010 |
| β-galactopyranosyl-1,3-arabinose | 95.55 | 586 | 0.032 | 0.007 |
| Glucose | 96.68 | 591 | 0.035 | -0.014 |
| D-Trehalose | 87.99 | 599 | 0.031 | -0.006 |
| D-Trehalose | 87.96 | 601 | 0.048 | -0.060 |
| Lactose | 82.13 | 605 | 0.054 | -0.025 |
| Adenosine | 92.28 | 612 | 0.018 | 0.060 |

**Table S5. List of 29 identified metabolites used to draw the metabolic pathways map shown in Fig. 4.** In this table are indicated relativized metabolite values of M4***-****Δgnd* and M4***-****ΔiclR* mutants respect to the M4 mutant values.

| **Compound** | **Acronyms** | **Match Factor** | **Pathway** | **M4-*Δgnd*** | | **M4-*ΔiclR*** | |
| --- | --- | --- | --- | --- | --- | --- | --- |
|  |  |  |  | **24 h** | **48 h** | **24 h** | **48 h** |
| (*R*)-lactate | Lac | 98.75 | Methylglyoxal degradation | 0.171 | 0.246 | 0.255 | 0.490 |
| (*R*)-malate | Mal | 92.99 | TCA cycle | 0.163 | 0.262 | 0.857 | 1.243 |
| 1,3-bisphospho-D-glycerate | 1,3-BPG | 74.45 | Glycolysis | 0.213 | 0.168 | 1.155 | 0.288 |
| 2-phospho-D-glycerate | 2-PG | 79.42 | Glycolysis | 0.015 | 0.152 | 0.292 | 0.912 |
| 3-methyl-2-oxobutanoate | 3-Met-2-Ox | 95.98 | Amino acid Biosynthesis | 1.153 | 1.979 | 1.239 | 1.828 |
| 3-phenyl-2-oxopropanoate | 3-Phe-Pyr | 95.83 | Amino acid Biosynthesis | 0.501 | 0.579 | 0.352 | 0.666 |
| 3-phospho-D-glycerate | 3-PG | 89.28 | Glycolysis | 0.078 | 0.132 | 0.797 | 0.831 |
| 4-methyl-2-oxopentanoate | 4-Met,2-Ox | 87.32 | Amino acid Biosynthesis | 0.441 | 0.435 | 0.605 | 0.447 |
| Acetic acid | Ac | 65.66 | Acetate utilization and formation | 0.009 | 0.221 | 0.209 | 0.041 |
| β-D-fructose-1,6-biphosphate | F1,6-BF | 86.23 | Glycolysis | 0.805 | 0.280 | 7.467 | 0.893 |
| Citrate | Cit | 96.31 | TCA cycle | 0.310 | 0.320 | 0.770 | 1.710 |
| D-fructose | Fru | 97.33 | Glycolysis | 0.154 | 0.053 | 0.975 | 1.041 |
| D-galactose | Gal | 98.37 | Carbohydrates | 0.133 | 0.044 | 0.853 | 0.353 |
| D-glucose 6-phosphate | G6P | 87.83 | Glycolysis | 0.292 | 1.440 | 0.816 | 11.744 |
| D-mannitol | Mnl | 84.8 | Glycerol assimilation | 2.037 | 0.947 | 2.225 | 0.840 |
| D-mannose 6-phosphate | Man-6P | 80.88 | Carbohydrates | 0.166 | 0.412 | 0.942 | 2.401 |
| D-rhamnose | Rha | 98.45 | Carbohydrates | 0.233 | 0.340 | 0.615 | 0.651 |
| Fumarate | Fum | 95.71 | TCA cycle | 0.275 | 0.338 | 0.420 | 0.549 |
| Glycolate | Glyc | 91 | Carbohydrate degradation | 0.283 | 0.591 | 0.849 | 0.767 |
| L-alanine | Ala | 95.86 | Amino acid Biosynthesis | 0.278 | 0.790 | 1.165 | 1.908 |
| L-glutamate | Glu | 95.61 | Amino acid Biosynthesis | 0.370 | 0.180 | 0.840 | 0.840 |
| L-glutamine | Gln | 92.52 | Nitrogen Regulation | 0.749 | 0.908 | 1.152 | 1.059 |
| L-phenylalanine | Phe | 95.3 | Amino acid Biosynthesis | 0.351 | 0.562 | 0.785 | 1.266 |
| L-serine | Ser | 93.25 | Amino acid Biosynthesis | 0.179 | 0.793 | 0.439 | 1.587 |
| L-valine | Val | 99.65 | Amino acid Biosynthesis | 0.490 | 0.139 | 2.038 | 0.458 |
| Pyruvate | Pyr | 96.84 | Glycolysis | 0.376 | 0.499 | 0.760 | 1.071 |
| *sn*-Glycerol 3-phosphate | Gly-3P | 86.97 | Glycerol assimilation | 0.712 | 0.186 | 0.932 | 0.437 |
| Succinate | Suc | 95.17 | TCA cycle | 0.376 | 0.499 | 0.760 | 1.071 |
| Trehalose | Tre | 87.99 | Carbohydrates | 0.051 | 1.223 | 0.617 | 7.736 |

**Table S6. List of 26 identified compounds related N metabolism used for heat map analysis as shown in Fig. 5.** In this table are shown the metabolites not analysed in the flux C analysis (Fig. 4), and the average of three replicates of peak areas. These set of metabolites were selected in based of their match factor were higher than 65.

| **Compound** | **Abbrev.** | **Match Factor** | **Pathway** | **M4-gnd 24 h** | **M4-gnd 48 h** | **M4-iclR 24 h** | **M4-iclR 48 h** | **M4**  **24 h** | **M4**  **48 h** |
| --- | --- | --- | --- | --- | --- | --- | --- | --- | --- |
| 3-Methyl-2-oxobutanoate | 3-Met-2-Ox | 95.98 | Amino acid Biosynthesis | 302687 | 236098 | 290565 | 219476 | 340191 | 55912 |
| Adenine-D-ribose | Ad-D-R | 92.28 | Nucleoside and Nucleotide Biosynthesis | 72728 | 27019 | 262198 | 71536 | 244263 | 29026 |
| Aminomalonic acid | Ama | 92.52 | No Metabolite | 31362 | 6564 | 66553 | 19335 | 132376 | 11619 |
| Glutarate | Glut | 89.86 | Amino acid Biosynthesis | 245049.53 | 111585 | 1410855 | 636413 | 1231173 | 401290 |
| Hidroxylamine | Hydrox | 96.38 | Others cell reactions | 16392354 | 17868854 | 15328050 | 17165911 | 6419526 | 2782288 |
| L-alanine | Ala | 95.86 | Amino acid Biosynthesis | 68030 | 22731 | 256898 | 52131 | 372505 | 101852 |
| L-ascorbic acid | Ascb | 86.14 | Carboxylate degradation | 203172 | 49445 | 577124 | 468431 | 27257518 | 10674779 |
| L-cysteinylglycine | Cys-glyc | 91.03 | Others cell reactions | 824993 | 496861 | 2208566 | 3227253 | 394853 | 349629 |
| L-glutamic acid | Glu | 95.61 | Amino acid Biosynthesis | 9272301 | 1898209 | 21545692 | 8753078 | 3541394 | 1724645 |
| L-glutamine | Gln | 92.52 | Nitrogen Regulation | 291003 | 303093 | 433399 | 358772 | 14713405 | 15921534 |
| L-isoleucine | ile | 98.49 | Amino acid Biosynthesis | 122163 | 35031 | 228572 | 158867 | 205291 | 32917 |
| L-methionine | Met | 97.66 | Amino acid Biosynthesis | 112680 | 31659 | 257713 | 240015 | 348494 | 119985 |
| L-Norvaline | Nval | 99.37 | No Metabolite | 2078064 | 123345 | 5055326 | 210257 | 3107175 | 147494 |
| L-phenylalanine | Phe | 95.3 | Amino acid Biosynthesis | 965738 | 193558 | 2146149 | 471766 | 256420 | 51153 |
| L-serine | Ser | 93.25 | Amino acid Biosynthesis | 74853 | 70697 | 133632 | 114260 | 5496052 | 4922906 |
| L-Valine | Val | 99.65 | Amino acid Biosynthesis | 179083 | 3569 | 493305 | 9471 | 20875132 | 19943039 |
| N-acetyl-D-glucosamine | Acgam | 83.86 | Cell structure Biosynthesis | 114477 | 78268 | 206088 | 203151 | 2746102 | 362960 |
| Nicotinamide | Nicot | 94.58 | Cofactor Biosynthesis | 2162945 | 1600824 | 5493560 | 2202253 | 81515278 | 39359531 |
| Orotate | Orot | 92.11 | Nucleoside and Nucleotide Biosynthesis | 6594883 | 4798704 | 8834459 | 4633073 | 16820258 | 15853127 |
| Phosphoric acid | Phos | 91.57 | Others cell reactions | 30795095 | 88605956 | 89672392 | 86947992 | 6268093 | 4640055 |
| Putrescine | ptrc | 98.46 | Amino acid degradation | 8568210 | 8386518 | 9817241 | 10237026 | 440327 | 101377 |
| Pyridine | Pyr | 98.01 | Cofactor Biosynthesis | 3497957 | 3218540 | 6539974 | 4472559 | 773351 | 756670 |
| Tryptamine | Tp | 99.53 | No Metabolite | 662479 | 680890 | 781438 | 730554 | 3081210 | 1717301 |
| Uracil | Ura | 91.66 | Nucleoside and nucleotide degradation | 1011988 | 706644 | 2942717 | 2909578 | 680409 | 942032 |
| Urea | Ure | 90.05 | Amino acid degradation | 803585 | 776125 | 684959 | 732455 | 338662 | 26085 |
| Xanthine | Xan | 90.04 | Nucleoside and nucleotide degradation | 112640 | 35627 | 397853 | 198501 | 413124 | 63340 |

**Table S7.** **List of the putative genes that codify the enzymes for the synthesis and transport of Mannitol and trehalose in *E. coli*.**

| **Enzyme Commission** | **Enzyme** | **Acronym** | **Source** |
| --- | --- | --- | --- |
| [3.1.3.22] | Predicted hydrolase mannitol-1-/sugar-/sorbitol-6-/2-deoxyglucose-6-phosphatase | YniC | KEGG |
|  | Predicted hydrolase or phosphatase mannitol-1-/sugar-/sorbitol-6-phosphatase | YfbT | KEGG |
|  | Hexitol phosphatase A | HxpA | EcoCyc |
|  | Hexitol phosphatase B | HxpB | EcoCyc |
|  | Sugar phosphatase | YbiV | EcoCyc |
|  | Sugar phosphatase | YidA | EcoCyc |
|  | Phosphosugar phosphatase | YigL | EcoCyc |
|  | Alkaline phosphatase | PhoA | EcoCyc |
| [2.7.1.197] | [mannitol-specific PTS enzyme IICB component CmtA](https://ecocyc.org/gene?orgid=ECOLI&id=CMTA-MONOMER) | CmtA | EcoCyc |
|  | M[annitol-specific PTS enzyme IIA component CmtB](https://ecocyc.org/gene?orgid=ECOLI&id=CMTB-MONOMER) | CmtB | EcoCyc |
|  | Mannitol-specific PTS enzyme II | MtlA | EcoCyc |
| [1.1.1.17] | Mannitol-1-phosphate 5-dehydrogenase | MtlD | EcoCyc |

**Figure S1**. PC1 and PC2 loadings plot obtained from PCA (Fig. 2) using all of the peaks detected by GC-MS analysis. All of the identified compounds related to *E. coli* metabolism are listed in Table S4.


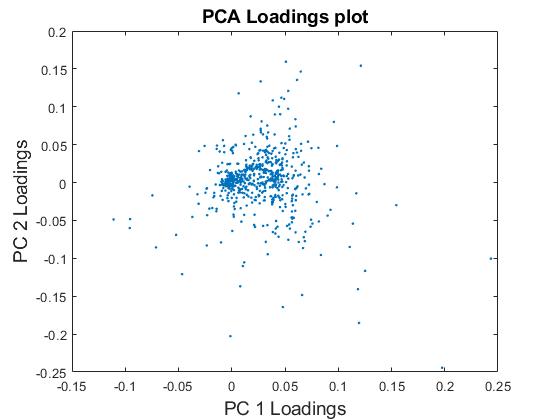


**Figure S2.** PCA scores plot of the pre-processed FT-IR spectral data of M4 mutant, M4-Δ*gnd* and M4-Δ*iclR* strains after 24 and 48 h since inoculation (*n* = 3) using PC1 and PC2 with 85.5% and 6.9% of total explained variance (TEV) respectively. PC1 loadings plot displayed relatively higher intensities for amide I (1655 cm^-1^, C=O stretching) and II (1545 cm^-1^, combination of C-N stretching and N‑H bending) bands of proteins and peptides, for samples on the far right of the PC1 axis, while exhibiting lower intensities for vibrational bands in the 900-1200 cm^‑1^ region, assigned to C‑O-C, C-O stretching dominated by ring vibrations in various polysaccharides (a). DF1 loadings averaged FT-IR spectra of the three strains at 24 h and 48 h that agreed with the PCA findings, where the M4-*ΔiclR* strain clearly displayed higher intensities for amide I and II, and significantly lower intensities for all the vibrations in the 900-1200 cm^-1^ region (b). Averaged FT-IR spectra dendrogram of the three strains at 24 h and 48h (c).


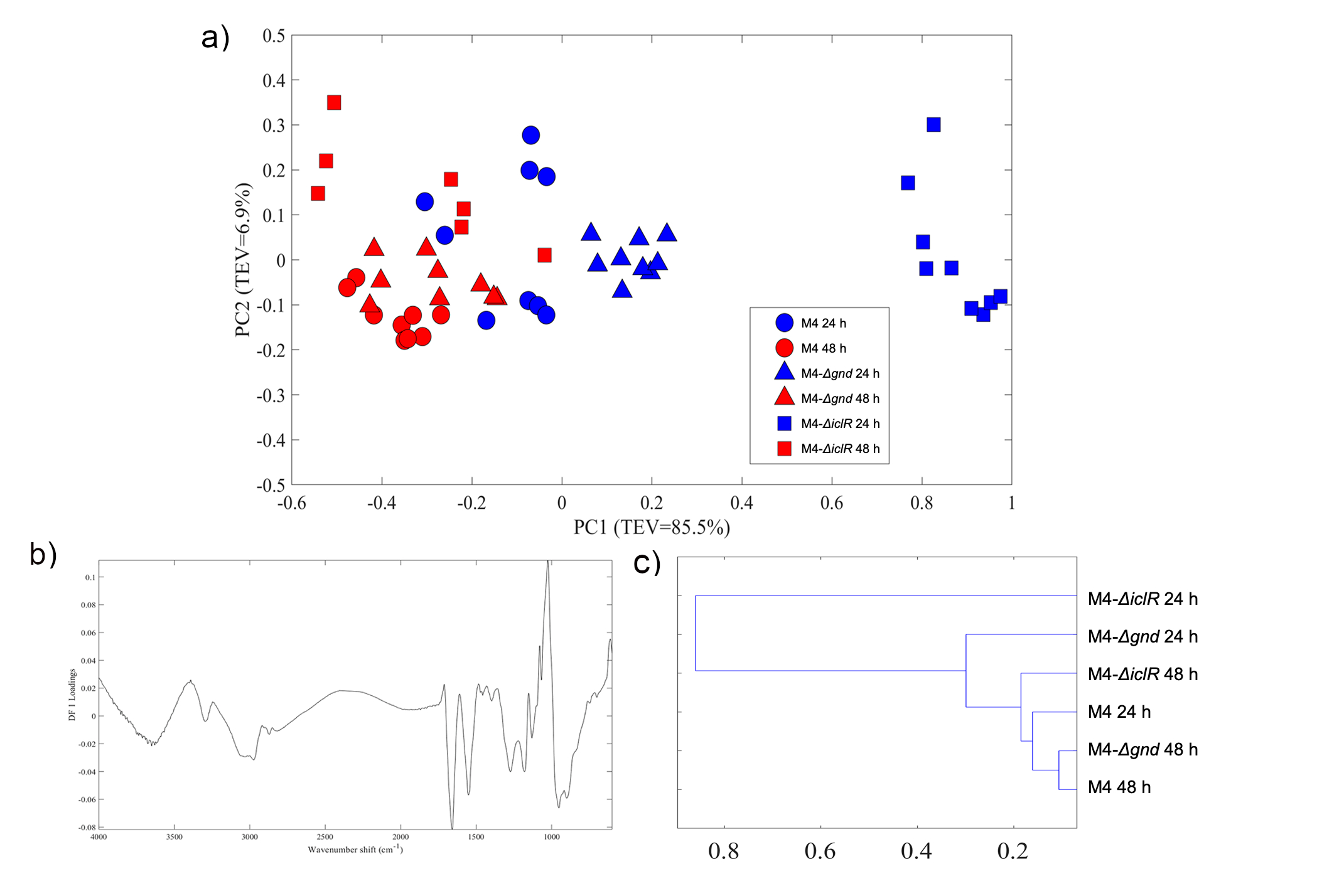


**Figure S3.** Pathway maps founded in KEGG database of Fructose and Mannose metabolism (a) and starch and sucrose metabolism (b).


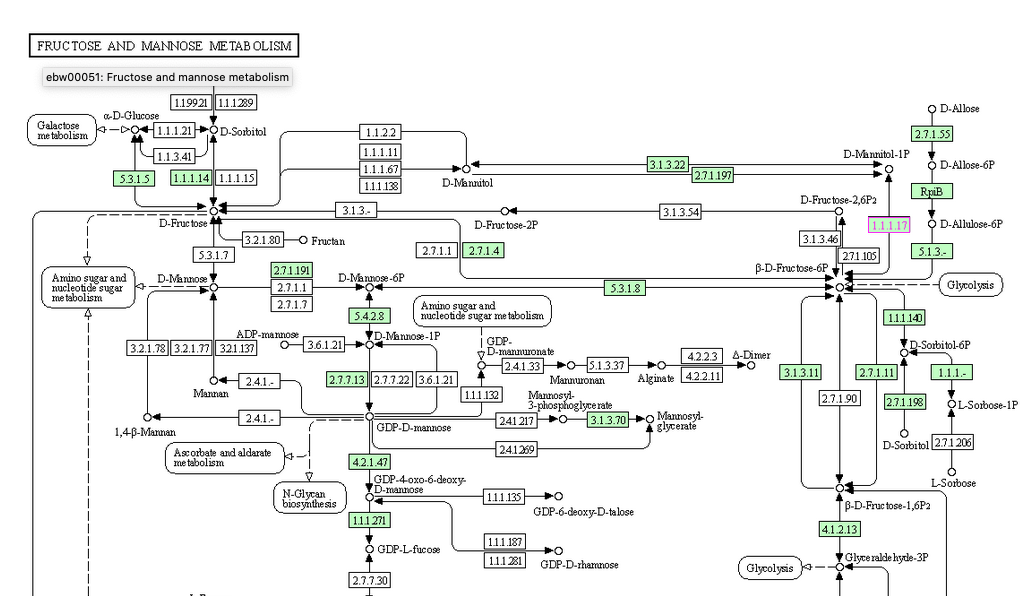


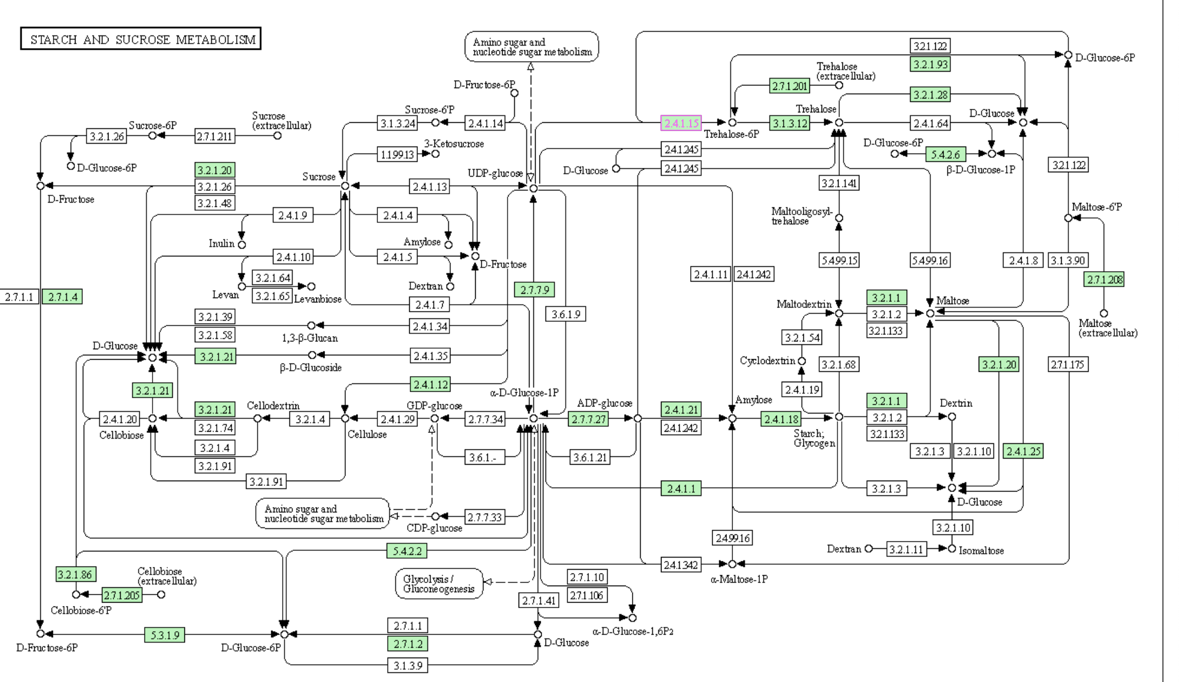


**Figure S4.** The ClustalW analysis of *mtlD* gene sequence respect to the predicted hydrolase and phosphatase enzyme’s genes (Table S7), show a high phylogram distance respect to the analyzed sequences (a); BLAST analysis of aminoacids sequences of Mannitol 1-phosphate 5-dehydrogenase (MtlD) (b) and trehalose synthase (OtsA) (c).


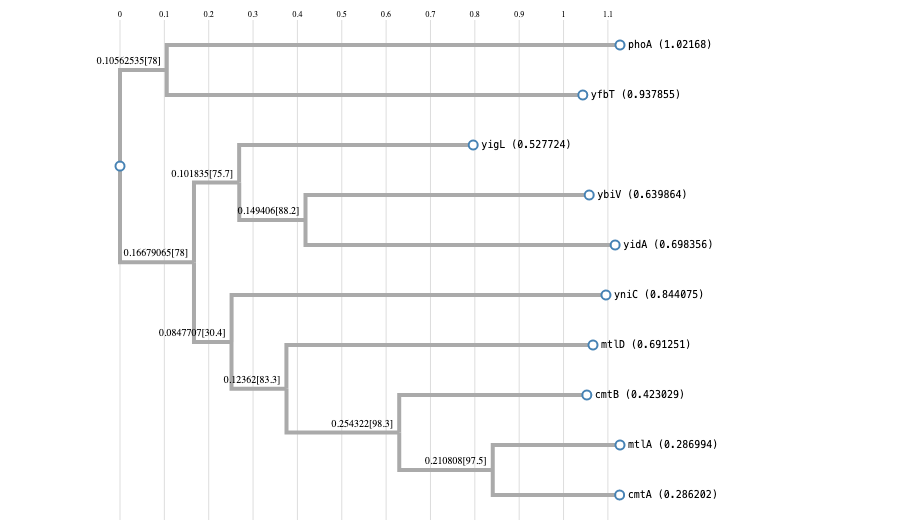


a)

b)


c)

**Figure S5**. Curves of pH (a and b) and acetic acid (mM) (c and d) in M4-*Δgnd*, M4-*ΔgndΔmtlD*, M4-*ΔiclR*, M4-*ΔiclRΔmtlD*, M4-*ΔiclRΔotsA* mutant strains*.* The time points selected were 0, 10, 24, 31 and 48 h.
